# SARS-CoV-2 infection and seropositivity among household contacts of laboratory confirmed cases of COVID-19 in residents of Delhi, India

**DOI:** 10.1016/j.pmedr.2024.102603

**Published:** 2024-01-10

**Authors:** Ayan Kumar Das, Farzana Islam, Yasir Alvi, Mridu Dudeja, Mohammad Ahmad, Anisur Rahman, Sushovan Roy, Aamir, Maroof Ahmed

**Affiliations:** a Department of Microbiology, Hamdard Insititute of Medical Science & Research, Jamia Hamdard, New Delhi 110062, India; b Department of Community Medicine, Hamdard Insititute of Medical Science & Research, Jamia Hamdard, New Delhi 110062, India; c World Health Organization Country Office for India, R.K. Khanna Tennis Stadium, 1, Africa Avenue, Safdarjung Enclave, New Delhi 110029, India

**Keywords:** SARS-CoV-2, Household transmission, Secondary infection rate, RT-PCR, Seroconversion, Asymptomatic infection

## Abstract

The transmission of respiratory pathogens, including SARS-CoV-2, is often facilitated through household contact. To better understand the transmission rate of COVID-19 among households and factors that affect viral clearance and seroconversion, a case-ascertained community-based prospective study was conducted between December 2020 and June 2021 on the urban population of the national capital region of India. The study collected nasopharyngeal swabs for SARS-CoV-2 RT-PCR on the 1st, 7th, 14th, and 28th day, and blood samples for antibody detection on the 1st, 14th, and 28th day from household contacts (HCs) of laboratory-confirmed COVID-19 cases. The study monitored the demographic data, symptoms, and outcomes of 417 participants, including 99 index cases and 318 contacts, for a period of 28 days. The results of the study showed that SARS-CoV-2 was easily spread within households, with a secondary infection rate of 44.3 %. In fact, almost 70 % of the contacts got infected within 1–2 days of identification of the index case, while 34 % remained asymptomatic. Sero-conversion was found in 35.6 % of the participants while 22.9 % did not produce antibodies after 28 days of infection. The study also revealed that females, spouses, older members, and primary care providers were at higher risk of getting infected in a home setting. However, approximately one-third of individuals in the younger age group managed to avoid infection. The study demonstrated that most infected individuals became RT-PCR negative within two weeks, although viral clearance was delayed in older patients and those with lower cycle threshold values in RT-PCR.

## 1 Introduction

The COVID-19 pandemic is arguably the biggest healthcare crisis that the world has faced in modern history. It has resulted in significant loss of life and health worldwide, as well as significant economic losses, particularly in developing countries like India. During the early part of the pandemic, there was confusion regarding transmission dynamics and epidemiological behaviour of the virus which made it difficult to control its spread. Even now, there is still much to learn about the transmissibility pattern and other epidemiological factors. Household transmission is one of the critical settings for the spread of most respiratory pathogens, including SARS-CoV-2. The proximity between family members, the enclosed environment, and the relaxation of personal protective measures at home all facilitate the viral spread. With strict lockdown measures enforced worldwide, the rate of household transmission may have been greater than community transmission (Li et al., 2020).

The host-virus interaction is complex and the outcome varies between individuals. The infection rate for a particular setting is not always constant and may depend on many associated factors. While viral clearance happens quickly in some individuals, shedding continues for a prolonged duration in others (McKie et al., 2020). Identifying and understanding these variations is crucial in decoding the transmission proclivities of COVID-19.

To control the pandemic, countries have implemented strict strategies. However, these strategies need local modifications to be more effective, and this requires knowledge about how the infection spreads in different settings. In low and middle-income countries like India, factors such as overcrowding and living standards can affect the transmission of the infection within households. The purpose of this study is to observe how SARS-CoV-2 spreads within households in urban populations of the national capital region. The study will analyze the factors associated with the rate of infection, viral clearance, and seroconversion among household contacts of COVID-19-positive cases.

## 2 Material and methods

This prospective case-ascertained study was conducted at a tertiary care hospital in Southern part of Delhi, India, between December 2020 to June 2021. This study was part of the WHO Unity protocol (World Health Organization, 2021), and it focused on household transmission of SARS-CoV-2. The study participants were included from the community, and they were the household contacts (HCs) of Index cases detected by Real-time polymerase chain reaction (RT-PCR) on the same day of testing (Day 1) at the hospital laboratory or of positive cases listed for the day by the district authority. Index cases were defined as primary cases of SARS-CoV-2 infection identified through RT-PCR test. A ‘household contact’ was defined as any person who has resided in the same household (or other closed setting like a hostel room) as a confirmed COVID-19 case.

The study excluded hospitalized index cases, households with more than 1 RT-PCR positive member (co-index cases), or symptomatic contacts on day 1 of the home visit. None of the study participants were vaccinated for SARS-CoV-2. The sample size was determined using the Schwartz formula, considering 0.5 as the secondary attack rate (Rathish et al., 2021, Dub et al., 2020). The calculated sample size was 100 households, and a sample collection of 400 participants was envisaged, considering 4–5 members per family. A household was defined as a group of two or more people residing in the same house (World Health Organization, 2021). The index cases were contacted telephonically, and verbal consent for a home visit for the collection of samples from household contact was obtained after explaining the purpose of the study. However, all hospitalized subjects were excluded from the study as it is difficult to determine the level of exposure to the contacts in such cases.

Prior to conducting home visits, all necessary permissions were obtained from district and state authorities. A team comprising of two laboratory technicians, one for data collection and the other for sample collection, was recruited and re-trained in standard and transmission-based precautions, and COVID-appropriate behaviour, as per Government of India/Indian Council of Medical Research (ICMR) protocols (Infection Prevention and Control (IPC) for COVID-19, Ministry of Health and Family Welfare, 2019). Data and specimen collection from the index cases and household contacts were done on the day of enrolment (Day 1), followed by home visits on Day 7, Day 14, and Day 28. Written consent was explicitly obtained from both the cases and the household contacts upon visiting the household. Demographic data, relationship with the index case, present symptoms, history of previous infection, and all other related data were collected through structured questionnaires. Each participant was provided with a symptom diary to record any symptoms manifesting within 28 days of the study period.

### 2.1 Specimen collection and transport

All baseline respiratory (nasopharyngeal and oropharyngeal swabs) and serum samples were collected from confirmed cases and their HCs according to GOI India's guidelines (Ministry of Health and Family Welfare, 2019). Respiratory samples were taken on Days 1, 7, 14, and 28, while paired blood sera were collected on Day 1, 14, and 28 for detection of anti-SARS-CoV-2 antibodies. The collection was carried out between 9:00 AM to 4:00 PM on designated days by a trained technician who visited pre-informed homes. The technician wore appropriate personal protective equipment (PPE) during sample collection. All samples were transported to the laboratory as quickly as possible to maintain a cold chain.

### 2.2 Laboratory analysis

#### 2.2.1 Detection of SARS-CoV-2 RNA

SARS-CoV-2 RNA was detected in the nasopharyngeal and oropharyngeal (NP/OP) swabs collected from the subjects by RT-PCR following guidelines laid down by ICMR (ICMR-National Institute of Virology (ICMR-NIV), 2019). The swabs collected in viral transport medium (VTM) were processed at the Biosafety level 2 molecular biology laboratory following all the safety protocols. The Viral RNA was extracted from the VTM using QIAamp Viral RNA mini kit, Qiagen, USA, following the kit protocol. Extracted RNA was further analyzed on the same day and was subjected to RT-PCR using COVIWOK RT-PCR Kit, SNP technologies, Turkey. The kit detects the N gene and the RdRp Gene of SARS-CoV-2. Both the extraction and RT-PCR kit are approved by ICMR. The assay was run in the Agilent MX3005P RT-PCR system. The cycle threshold (Ct) value cut-off for both the viral genes and the internal control was considered as < 35, as mandated by ICMR. The results were communicated and uploaded into the ICMR portal. The samples were stored for future reference for the next 6 months at −80 °C.

**2.2.2. Anti-SARS-CoV-2-total antibody detection:** Anti-SARS-CoV-2-total antibody was detected in patient samples using the Wantai SARS-CoV-2-Ab ELISA kit (Li et al., 2021). The kit detects total antibodies against the SARS-CoV-2 virus and is based on the principle of a two-step incubation antigen “sandwich” enzyme immunoassay. Briefly, 100 ml of the patient’s serum is added to polystyrene microwell strips pre-coated with recombinant SARS-CoV-2 antigen. Three wells are marked as negative calibrators and 2 wells as positive calibrators. 50 ml of negative and positive calibrators are added to respective wells and the plate is incubated at 370C for 30 min. Post incubation the wells were washed 5 times with diluted wash buffer. 100 μl of HRP-Conjugate was then added to each well and the plate was incubated at 370C for 30 min. The wells were washed again washed 5 times and 50 μl of Chromogen Solution A and then 50 μl of Chromogen Solution B was added into each well. The plate was then incubated at 37 °C for 15 min in dark. 50 μl of Stop Solution was added into each well and mixed gently. Absorbance was measured using PR4100 microplate reader, Bio-Rad, USA (dual filter) with reference wavelength at 600 ∼ 650 nm. The cut-off value (C.O.) was calculated as C.O = Nc + 0.16 (Nc = the mean absorbance value for three negative calibrators). The biomedical wastes generated during the process were disposed of according to biomedical waste disposal protocol. The tested serum samples were stored at −800C with proper labelling.

### 2.3 Statistics analysis

The data was tabulated using MS Excel and analyzed with IBM Statistical Package for Social Sciences (SPSS) Version 25, as well as an online statistical tool: https://epitools.ausvet.com.au. Demographic data was presented as the mean value ± standard deviation (SD). The prevalence percentage and 95 % confidence interval (CI) were calculated. The association between categorical variables was analyzed using the χ2 test, while for continuous variables with a dichotomous outcome, an unpaired *t*-test was used. In case of more than two outcomes, a one-way ANOVA was used to assess the level of significance. The level of significance was considered to be p < 0.05.

### 2.4 Ethical considerations

The study was part of WHO unity protocol approved by the WHO Research Ethics Review Committee (ERC0003356). Ethical clearance was also obtained from and Institutional Ethical committee (IEC-2020/15).

## 3 Result

The study included 99 index cases who met the selection criteria and agreed to participate. These cases were initially identified from the hospital's COVID-19 testing centre and through district authorities. The average household size of the selected index cases was 4.48 ± 1.96. Out of the 345 HCs approached, 318 met the inclusion criteria and agreed to participate in the analysis.

During the study, it was observed that 34 % of individuals who tested positive for RT-PCR did not exhibit any symptoms during the entire course of 28 days. The average age of female asymptomatic positive cases was higher than that of male cases, at 40.53 ± 21.3 and 31.47 ± 21.51 respectively. However, no significant difference was observed in terms of gender and age between symptomatic and asymptomatic RT-PCR positive cases, as confirmed by an unpaired *t*-test (t-value: −0.40, p-value: 0.344).

In Table 1, the demographic details of the participants and the rate of positivity among household contacts are presented. The index cases had a mean age of 38.73 ± 14.38 years, while their contacts had a mean age of 33.28 ± 19.43 years. It was observed that the HCs who became RT-PCR positive had a significantly higher mean age than those who remained RT-PCR negative (p = 0.017). The HCs were divided into various age groups, and while the highest RT-PCR positivity was observed among those aged 31–50, the finding was not statistically significant. There was a comparable number of male and female participants in the study. However, female participants showed a significantly higher RT-PCR positivity compared to men (p = 0.0006).

**Table 1:** Demographic details of participants and rate of SARS-CoV-2 positivity among the household contacts of index cases from Southern Delhi, India (Dec 2020–Jun 2021).

	**Index cases**	**Household Contacts (HC)**	**RT-PCR Negative HC**	**RT-PCR Positive HC**	**Positivity rate in HC**	**95 % CI**	**P-value (significant < 0.05)**
**N**	99	318	177	141	44.3 %	38.8–49.9	**0.017** (t value = − 2.12)
**Age (years)** (mean ± SD)	38.7 ± 14.38	33.2 ± 19.4	31.2 + 18.5	36.0 ± 20.0			
**Positivity in different age groups in years**							
0–15	03	71	46	25	35.2 %	24.2–47.4	0.25 (*χ^2^ = 0.18*)
16–30	28	89	50	39	43.8 %	33.3–54.7	
31–50	46	82	40	42	51.2 %	39.9–62.4	
51-above	22	76	41	35	46.0 %	34.5–57.8	
**Gender**							
Male	59	156	102	54	34.6 %	27.6–42.3	**0 0.0006** (*χ^2^ = 11.7*)
Female	40	162	75	87	53.7 %	46.0–61.2	
**Relation of HC with Index case**							
Spouse		62	28	34	54.8 %	42.5–66.5	
In law		28	14	14	50.0 %	32.6–67.3	**0.03** (*χ^2^ = 13.6*)
Maid/Care taker		22	11	11	50.0 %	23.5–57.5	
Parents/		53	31	22	41.5 %	29.2–54.9	
Siblings		30	17	13	43.3 %	27.3–60.8	
Son/daughter		92	50	42	45.6 %	35.8–55.8	
Others*		31	26	5	16.1 %	07.0–32.6	

*(uncle, aunt, friend/roommate, nephew, niece, grand children).

The study analyzed the relationship between HCs and the index case. The infection rate was nearly the same among first-degree relatives and caretakers/maids, but significantly lower among second-degree relatives, friends, and roommates.

The RT-PCR positivity rate among the HCs was categorized based on the time of the first positive report for each participant, as shown in Table 2. The analysis revealed that over 70 % of the infected contacts tested positive within 0–2 days of the detection of the index case. Around 20 % tested positive by the 7th day, while 4.9 % became positive between the 8th and 14th day. Only 4.2 % of the participants tested positive beyond the 15th day. The day/week of RT-PCR positivity among HCs was also compared based on different factors such as age groups, gender, relationship with index cases, and co-morbidities. However, no significant difference was observed in the positivity rate among the different groups.

**Table 2:** Time taken for household contacts to become RT-PCR positive for SARS-CoV-2 after identification of index cases in Southern Delhi, India (Dec 2020–Jun 2021).

	**RT-PCR positive HC subjects**				**P value**
	**in 0**–2 days*	**Within 7 days**	**Between 8th–14th day**	**Between 15th–28th day**	
**n = 141**	100	28	07	06	0.59 (*F value = 0.62*)**
**Age (years)** **(mean ± SD)**	36.9 ± 19.9	31.96 ± 19.7	39.5 ± 23.6	31.1 ± 23.4	
**As per age group**					
00–15	15(15 %)	07(25 %)	01(14.2 %)	02(33.3 %)	0.87 (*χ^2^ = 4.5*)
16–30	27(27 %)	09(32.1 %)	03(42.8 %)	01(16.6 %)	
31–50	32(32 %)	07(25 %)	02(28.5 %)	02(33.3 %)	
51-above	26(26 %)	05(17.8 %)	01(14.2 %)	01(16.6 %)	
**Gender**					
Male	38(38 %)	12(42.8 %)	02(28.5 %)	03(50 %)	0.83 (*χ^2^ = 0.84*)
Female	62(62 %)	16(57.1 %)	05(71.4 %)	03(50 %)	
**Relation of HC with Index case**					
Spouse	23(23 %)	08(28.5 %)	02(28.5 %)	01(16.6 %)	0.16 (*χ^2^ = 23.8*)
In law	06(06 %)	03(10.7 %)	03(42.8 %)	02(33.3 %)	
Maid/Care taker	07(07 %)	04(14.2 %)	–	–	
Parents/	12(12 %)	02(07.1 %)	01(14.2 %)	01(16.6 %)	
Siblings	11(11 %)	02(07.1 %)	–	–	
Son/daughter	32(32 %)	08(28.5 %)	–	02(33.3 %)	
Others	03(03 %)	01(03.5 %)	01(14.2 %)	–	
**Comorbidities**	12(12 %)	04(14.2 %)	02(28.5 %)	2(33.3 %)	0.33(*χ^2^ = 3.3*)

*Day Zero: day when index case is detected positive; **One-way ANNOVA.

Table 3 provides a comparison of RT-PCR-positive household contacts (HCs) in terms of the duration required for them to turn RT-PCR negative. More than half of the HH contacts were able to achieve a negative status within a week, while 37.5 % took up to two weeks, and only 10.6 % remained RT-PCR positive beyond the second week. One participant remained positive on the 28th day after their first RT-PCR positive report. It was found that the mean age of participants who turned negative within a week was significantly lower than those who took longer (p = 0.01). There was no correlation between the duration required to turn RT-PCR negative and gender or co-morbidities.

**Table 3:** Duration for SARS-CoV-2 infected household contacts to become RT-PCR negative in Southern Delhi, India (Dec 2020–Jun 2021).

	**Time taken to turn RT-PCR negative**			**P value**
	**In ≤ 1 week**	**In ≤ 2 week**	In ≤ 3 weeks*	
**n = 141**	72	53	15	0.01 (*F value = 4.4*)**
**Age (years)** **(mean ± SD)**	33.2 **±** 18.5	35.15 ± 20.5	49.9 ± 22.1	
**As per age group**				
00–15	16(22.2 %)	07(13.2 %)	02(13.3 %)	0.06 (*χ^2^ = 11.9*)
16–30	18(25 %)	20(37.7 %	01(06.6 %)	
31–50	25(34.7 %)	12(22.6 %)	05(33.3 %)	
51-above	13(18.0 %)	14(26.4 %)	07(46.6 %)	
**Gender**				
Male	27(37.5 %)	22(41.5 %)	06(40 %)	0.9 (*χ^2^ = 0.2*)
female	45(62.5 %)	31(58.4 %)	09(60 %)	
**Comorbidities**	12(16.6 %)	04(07.5 %)	04(26.6 %)	0.12 (*χ^2^ = 4.17*)

*1 participant was found positive at 28th day; **One-way ANNOVA.

**Fig. 1:**
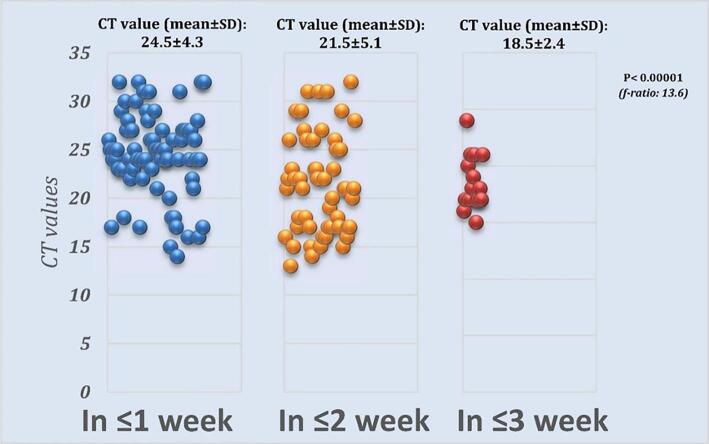
Ct values of first SARS-CoV-2 RT-PCR positive report of individuals grouped as per time taken to turn negative in Southern Delhi, India (Dec 2020–Jun 2021).

Fig. 1 shows a scatterplot of Ct values from the initial RT-PCR reports of all positive household cases. The data reveals that the average Ct value of individuals who took more than two weeks but less than three weeks to become RT-PCR negative was significantly lower than those who tested negative in less than two weeks (p < 0.00001).

A total of 277 HCs were analyzed for the presence of anti-SARS-CoV-2 antibodies, thrice during the study period (Table 4). Among those tested, 38.2 % of participants were positive on day zero of testing. Among those who were negative on days 0–2, 64.3 % remained negative until the 28th day, while 35.6 % turned positive within 28 days. By the 14th day, 19.8 % were positive, and the remaining 15.7 % turned positive by the 28th day. The study also found that the mean age of those who remained negative was lower than those who tested positive **(p = 0.00311)**.

**Table 4:** Proportion of household contacts of index cases with SARS-CoV-2 sero-positivity in Southern Delhi, India (Dec 2020–Jun 2021).

	**Household** **Contacts (HC) tested**	**HC sero-negative on day 0**–**2**	**HC sero-positive on day 0**–**2**	**HC who turned sero-Positive in later weeks**	**HC who remained sero negative**	**Sero conversion**	**95 % CI**	**P-value (significant < 0.05)**
**n**	277	171	106	61	110	35.6 %	28.8–43.3	**0.00311** (F = 5.89) *
**Age**(mean ± SD)	35.5 ± 18.4	33.9 ± 19.1	38.0 ± 16.8	39.3 ± 19.6	30.9 ± 18.33			
**Positivity in different age groups in years**								
00–15	46	39	07	09	30	23.0 %	12.6–38.3	0.12 (*χ^2^ = 5.8*)
16–30	81	45	36	14	31	31.1 %	19.5–45.6	
31–50	79	44	35	18	26	40.9 %	27.6–55.5	
51-above	71	43	28	20	23	46.5 %	32.5–61.0	
**Gender**								
Male	133	78	55	25	53	32.0 %	22.7–43.3	0.36 (*χ^2^ = 0.8*)
female	144	93	51	36	57	38.7 %	29,4–48.8	

*One-way ANNOVA.

Out of the people who tested positive for antibodies on day 1, only 25.4 % also tested positive for SARS-CoV-2 using RT-PCR. Within the study period, 66.6 % of those who were seropositive did not get infected, while only 8.4 % tested positive for SARS-CoV-2 in subsequent weeks. In addition, out of the 99 index cases and 106 HCs who tested positive for SARS-CoV-2 using RT-PCR on the day of enrolment, 47 did not develop antibodies until the 28th day.

## 4 Discussion

The present study tried to identify the rate of viral spread among HCs and evaluate it with associated factors for a better understanding of transmission dynamics. After the identification of index cases, they were approached for initial consent and enrolment followed by enrolment of family members. A total of 417 participants from 99 households (99 index cases and 318 contacts) completed the study and were monitored for 4 weeks for symptoms, SARS-CoV-2 RT-PCR, and antibody detection.

The average age of HCs who contracted the infection (RT-PCR positive), was significantly higher (p = 0.017) than those who remained negative even after 4 weeks of exposure. Highest positivity was observed among the age group of 31–50, followed by age group of 51 and above. Studies conducted around the globe have shown that the household secondary infection rate increases with the age of contacts (Goldstein et al., 2020, Park et al., 2020) and this holds for the Indian scenario also. Many factors like the affinity of the ACE2 receptor on an epithelial cell, lifestyle habits, and comorbidities have been found associated with an increase in susceptibility to SARS-CoV-2 with age (Zimmermann and Curtis, 2021). The study included comparable number of numbers of male and female HCs, but observed that significantly higher percentage of female participants became infected compared to males. While many studies did not notice any relation between household transmission and gender, several others have reported higher infection rates among females (Ogata et al., 2022, BARDHAR, 2022). The finding seems logical to the Indian scenario where a considerable number of the female are housewives and primary caregivers to the diseased and hence are more exposed. The observation regarding higher susceptibility to infection among females and older individuals is crucial for understanding disease transmission dynamics in households and should be considered when formulating guidelines for community transmission of diseases such as COVID-19.

The study examined the correlation between index cases and their HCs who tested positive for RT-PCR. The results showed that spouses had the highest rate of infection, which is consistent with findings from other studies (Madewell et al., 2020). This may be due to factors such as sharing a room and prolonged direct exposure. Previous research has also shown that household transmission between spouses is higher for SARS-CoV-1 and H1N1 infections (Pang et al., 2003.JAMA. 2003; Pang et al., 2011). On the other hand, household infection rates were found to be significantly lower among non-1st-degree relatives, friends, and roommates compared to first-degree relatives and maids/caretakers (p = 0.03). This could be due to the fact that these contacts have less exposure time and proximity to the index case.

Asymptomatic infection is one of the major factors that positively influence household transmission of the disease. Studies conducted worldwide have reported 30–40 % asymptomatic carriage of the virus (Oran and Topol, 2020, Ma et al., 2021). In the present study, over one-third of the infected HCs remained asymptomatic during the course of the infection. Asymptomatic cases tend to spread the disease more aggressively, as those infected and their contacts may follow preventive measures less strictly when compared to symptomatic cases. The study underscores the need for COVID-appropriate behaviour within households at all times.

The study provided a unique perspective on disease transmission within households in India by monitoring the HCs for 28 days in terms of symptomology and RT-PCR testing. The findings showed that the virus spreads quickly among HCs, with nearly two-thirds of them turning RT-PCR positive within 0–2 days of identification of the index case. Although each index case was counselled about COVID-19 appropriate behaviour and self-isolation and was followed up with, it was found that it is extremely difficult to prevent household spread. This is mainly due to the fact that by the time the index case shows symptoms or is detected positive, many of the HCs are already in the incubation period. Although the percentage is relatively lower, HCs may still get infected even after the third week. In a similar study conducted in the USA, with a secondary attack rate of 60 %, two-thirds of HCs were already RT-PCR positive by the time of enrolment (Cerami et al., 2021). The study did not find any significant correlations between the time taken for secondary infection and age, gender, or co-morbidities of HCs.

Our research revealed that more than half of the individuals who were infected with the virus turned out to be negative on RT-PCR tests within a week. Few individuals remained RT-PCR positive beyond 2 weeks and only one individual was RT-PCR positive on day 28. However, in most cases, even though the assay detects viral RNA in the nares or oral cavity, the subjects are usually cured and no longer contagious. Infectious or replication-competent viruses are isolated within two weeks of most infections unless the individual is immunocompromised (Owusu, 2021). We also found that younger individuals had a faster viral clearance rate, which is an important observation and can help decide the isolation time for different age groups in case of COVID-19 and similar infections. Some studies have shown that the presence of co-morbidities like hypertension, chronic heart disease, etc. can have a negative effect on viral clearance (Shu et al., 2021). However, in our study, we did not observe any such association.

The Ct value of the RT-PCR test indirectly estimates the viral load by indicating the number of amplification cycles at which the fluorescence signal crosses the threshold. A lower Ct value indicates a higher viral load (Owusu, 2021). After analysing the Ct values of the initial tests of all the positive patients, we found a direct correlation between these values and the time it took for the virus to clear out of the system in weeks. Although a lower Ct value may not indicate the severity of the disease, as we found that many such cases remained asymptomatic, we noticed that a higher viral load during the early stages of the infection was linked to a delay in the clearance of the virus from the system. Zhao et al from China had previously noted this correlation (Zhao et al., 2022). This finding can help in better understanding the pathophysiology of SARS-CoV-2 infection.

Few interesting observations were made in terms of seropositivity among the non-vaccinated population. More than one-third of the HCs tested were carrying anti-SARS-CoV-2 antibodies at the time of enrolment indicating they have already been exposed or infected previously from sources other than the present index case of their household. The nearly same number of participants escaped sero-conversion during the 28 days of observation, and their mean age was significantly lower than those who were detected positive for SARS-CoV-2 antibodies. Better adherence to COVID-19 appropriate behaviour at home or lesser involvement in patient care by these HCs or any other factor(s) can be responsible for such findings and may require further analysis.

22.9 % of RT-PCR-positive participants remained non-seroconverted till the 28th day of infection. While studies have linked older age and co-morbidities with non-seroconversion (Masiá et al., 2021), no such association was observed in the present study. Viral load, age, the immune and hormonal status of the patient, or even the sensitivity of the antibody detection method or kit may play a crucial role in determining sero-conversion (Zhang et al., 2020).

The study had some limitations. The participants were chosen through convenience sampling, with only those who tested positive during their visit to a hospital or testing centre being included, limiting the generalizability of the results. The transmission dynamics in hospitalized cases were difficult to determine as they were separated from their families, so those cases were excluded from the study. However, this exclusion meant that the study did not analyze the transmission dynamics in more severe primary cases.

## 5 Conclusion

The study highlights how easily the SARS-CoV-2 virus can spread within households. It is more likely for females, spouses, older family members, and primary care providers to get infected. The virus spreads rapidly within the household due to high infectivity before the onset of symptoms and also because a large number of infected individuals remain completely asymptomatic. Interestingly, around one-third of younger people in a household can remain uninfected. Most infected individuals test negative by RT-PCR within two weeks. However, older patients and those with lower Ct values may take longer to clear the virus.

## Funding source

This study was supported by the World Health Organization, under the UNITY Studies. [WHO reference: 2020/1055876-0, 2020].

## CRediT authorship contribution statement

**Ayan Kumar Das:** . **Farzana Islam:** Writing – review & editing, Resources, Project administration, Methodology, Funding acquisition, Formal analysis, Conceptualization. **Yasir Alvi:** Writing – review & editing, Validation, Software, Methodology, Formal analysis, Data curation. **Mridu Dudeja:** Writing – review & editing, Supervision, Project administration, Methodology, Funding acquisition, Formal analysis. **Mohammad Ahmad:** Writing – review & editing, Resources, Project administration, Funding acquisition, Formal analysis, Conceptualization. **Anisur Rahman:** Resources, Funding acquisition, Formal analysis, Data curation, Conceptualization. **Sushovan Roy:** . **Aamir:** . **Maroof Ahmed:** .

## Declaration of competing interest

The authors declare that they have no known competing financial interests or personal relationships that could have appeared to influence the work reported in this paper.

## Data Availability

Data will be made available on request.
