# Supplementary figures and images for: SARS-CoV-2 infection and seropositivity among household contacts of laboratory confirmed cases of COVID-19 in residents of Delhi, India
Source: Prev Med Rep. 2024 Jan 10;38:102603. doi: 10.1016/j.pmedr.2024.102603 (PMC10826300; doi:10.1016/j.pmedr.2024.102603)

Flowchart: characteristics of participants according to RT-PCR results and serostatus

**
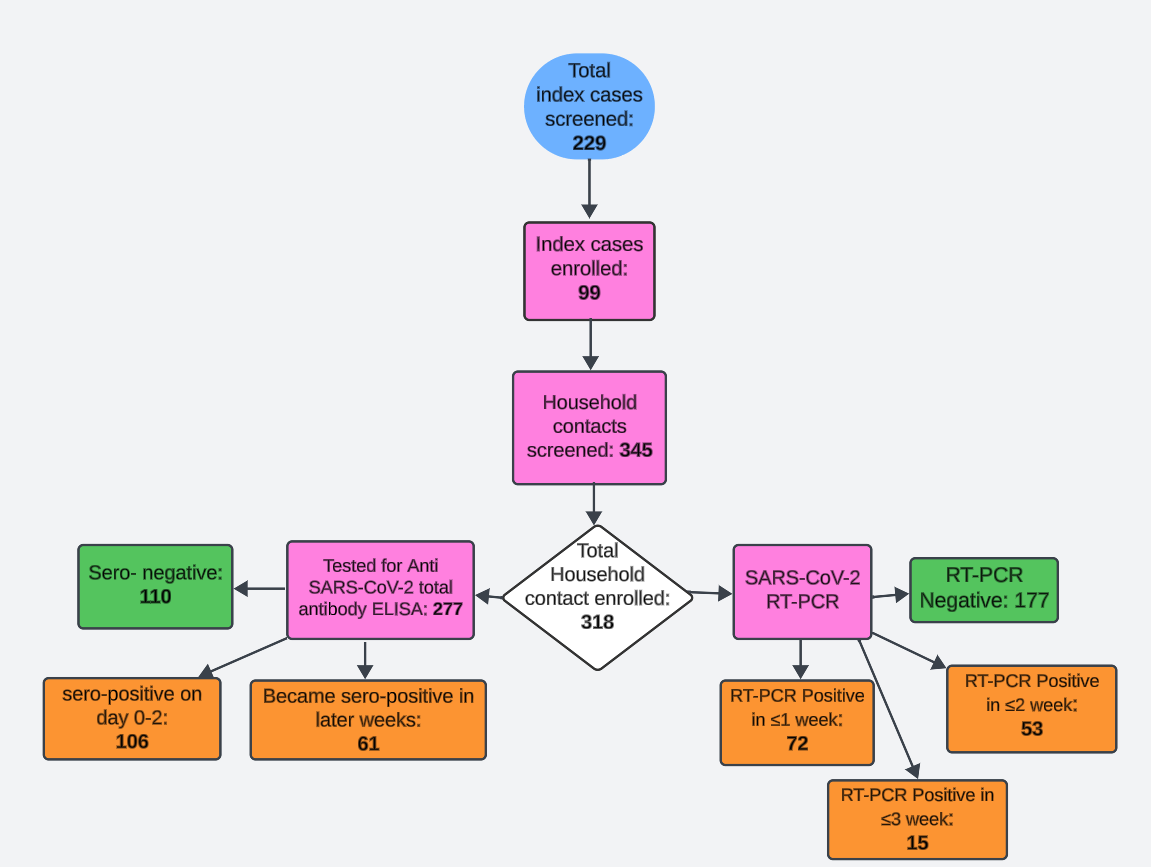
**

Supplement: Supplementary data 1 [file mmc1.docx]
